# Combined Transcranial Direct Current Stimulation and Virtual Reality in Healthy Populations: A Systematic Review of Evidence, Limitations, and Methodological Challenges

**DOI:** 10.3390/bioengineering13080883

**Published:** 2026-07-31

**Authors:** Chiara Milasi, Maria Grazia Maggio, Giuseppe Perrotti, Paola Barbuto, Marina Barberio, Alfredo Albertini, Nicola Tallarico, Federico Rocca, Marianna Contrada, Francesca Gallivanone, Andrea Gaggioli, Rocco Salvatore Calabrò, Cristina Segura-Garcia, Domenico Bosco, Antonio Cerasa

**Affiliations:** 1 Institute of Bioimaging and Complex Biological Systems (IBSBC), National Research Council of Italy (CNR), 88100 Catanzaro, Italy; chiaramilasi@cnr.it (C.M.); perrottigvet@gmail.com (G.P.); federico.rocca@cnr.it (F.R.); 2 IRCCS Centro Neurolesi Bonino-Pulejo, Via Palermo, SS 113, C. da Casazza, 98124 Messina, Italy; mariagraziamay@gmail.com (M.G.M.); roccos.calabro@irccsme.it (R.S.C.); 3 Department of Medical and Surgical Sciences, University Magna Græcia of Catanzaro, 88100 Catanzaro, Italy; paola.barbuto002@studenti.unicz.it (P.B.); marina.barberio@studenti.unicz.it (M.B.); alfredo.albertini@studenti.unicz.it (A.A.); nicola.tallarico@studenti.unicz.it (N.T.); 4 S. Anna Institute, 88900 Crotone, Italy; mariannacontrada@gmail.com; 5 Institute of Bioimaging and Complex Biological Systems (IBSBC), National Research Council of Italy (CNR), 20090 Milan, Italy; francesca.gallivanone@cnr.it; 6 Research Center in Communication Psychology (PSICOM), Università Cattolica del Sacro Cuore, 20123 Milan, Italy; andrea.gaggioli@unicatt.it; 7 Outpatient Unit for Clinical Research and Treatment of Eating Disorders, University Hospital Renato Dulbecco, 88100 Catanzaro, Italy; segura@unicz.it; 8 Psychiatry Unit, Department of Health Sciences, University Magna Græcia of Catanzaro, 88100 Catanzaro, Italy; 9 Institute of Neurology, Department of Neurosciences, Presidio Ospedaliero “Pugliese”, AOU “Renato Dulbecco”, 88100 Catanzaro, Italy; nico.bosco@libero.it

**Keywords:** virtual reality, transcranial direct current stimulation, healthy participants, emotional control, motor skills

## Abstract

Objectives: This systematic review examined studies combining transcranial direct current stimulation (tDCS) with virtual reality (VR) in healthy or non-clinical populations. The aim was to evaluate whether active tDCS provides additional benefits when delivered during VR-based tasks or interventions aimed at improving behavioral abilities. Methods: Following PRISMA guidelines, a comprehensive search of electronic databases (2000–September 2025) identified randomized and non-randomized studies employing simultaneous tDCS and VR in healthy individuals. Studies reporting psychological or cognitive quantitative outcomes were included. Risk of bias was assessed using RoB-2 and ROBINS-I tools. Results: Twelve studies met inclusion criteria. The included studies were highly heterogeneous in terms of sample size, VR systems, stimulation parameters, targeted cortical regions, outcome measures, and control conditions. Most designs compared active tDCS during VR with sham tDCS during the same VR exposure. Therefore, the available evidence mainly addresses whether tDCS adds value to VR-based procedures, rather than whether tDCS and VR exert independent or synergistic effects. Some studies reported favorable between-group effects on emotional control or skill acquisition. However, in other cognitive domains both active and sham/control VR groups improved, suggesting that VR training or exposure itself may have contributed substantially to the observed changes. Moreover, small sample sizes, multiple outcome testing, and limited correction for multiple comparisons reduce confidence in borderline findings. Conclusions: Current evidence suggests that active tDCS may incrementally enhance selected VR-based outcomes in healthy populations, particularly in emotional control and skill-learning domains. However, the literature does not yet support strong claims of synergistic interaction between tDCS and VR. Future studies should use adequately powered factorial designs including VR-only, tDCS-only, combined, and sham/no-intervention conditions to disentangle independent, additive, and interaction effects.

## 1. Introduction

Positive technologies (PTs) are designed to improve the quality of human experience by promoting well-being, engagement, and social connectedness [1]. They include tools such as virtual reality (VR), mobile applications, and wearable devices, and aim not only to reduce symptoms but also to enhance cognitive functioning and quality of life [2,3]. This broader focus makes PTs especially relevant for research in healthy populations. For instance, apps can deliver Positive Psychology Interventions and self-guided cognitive–behavioral therapy modules, while wearable and biofeedback devices can support personalized interventions through real-time monitoring of biobehavioral parameters [4,5,6,7].

Among PTs, VR has received particular attention because it offers immersive, controllable, and ecologically valid environments that can support emotional and psychological change [8]. In clinical research, VR has been applied in several psychopathological domains, including eating disorders [9], anxiety disorders [10] and post-traumatic stress disorder (PTSD) [11]. According to Kim & Kim [11] and Riva & Serino [8], interventions of this type frequently enable repeated exposures, a gradual modification of intensity, and a personalization customized to the patient’s demands, making VR a flexible adjunct or an alternative to traditional therapies. In a similar vein, people with PTSD can confront memories that would otherwise be impossible to evoke without taking undue risks thanks to the VR technique. Significant improvements in post-treatment symptoms and favorable acceptance ratings have also been documented in this setting [8,9,10,11]. Patients with specific phobias or social anxiety disorder can progressively and repeatedly approach fearful stimuli with Virtual Reality Exposure Therapy (VRET), which is the preferred treatment for tackling avoidance [12].

In the last few years, accumulating evidence suggests that combining positive technology (PT) applications with non-invasive brain stimulation (NIBS) may enhance clinical outcomes in several neurological and psychiatric disorders [13,14]. Among NIBS approaches, transcranial direct current stimulation (tDCS) is particularly attractive because it relies on relatively simple, low-cost equipment and offers greater portability and potential wearability, while generally being less burdensome for participants—features that support broader, scalable applications. tDCS delivers a low, constant current (typically 1–2 mA) between scalp electrodes for several minutes; the resulting intracerebral currents slightly polarize neuronal membranes and thereby shift their excitability by influencing synaptic plasticity [15]. In patients with PTSD, van ’t Wout-Frank et al. [16] demonstrated that combined treatment with anodal tDCS over the ventromedial prefrontal cortex and warzone VR exposure reduces pathological arousal. In this randomized control trial (RCT) study, patients underwent combined 2 mA anodal tDCS or sham sessions during VR exposure for 12 weeks, demonstrating accelerated psychophysiological habituation to immersive stressing events compared with sham tDCS. In patients with binge eating disorders, Max et al. [17] investigated the effects of combining verum or sham tDCS over the dorsolateral prefrontal cortex (DLPFC) with a VR-based food interaction task. In this study, a significant reduction in binge eating frequency over time was detected; however, this significance did not survive a direct comparison between verum or sham tDCS. In anxiety disorders, there are many studies combining these two methods generally in phobic contexts [18]. For instance, McDonald et al. [19] used anodal tDCS for stimulating neural areas associated with safety learning (the medial prefrontal cortex (mPFC)) during an immersive condition of social speaking. Individuals with pathological levels of social anxiety showed reduced symptoms after treatment facilitating safety signaling during exposure therapy. In the neurological domain, tDCS has extensively been used in combination with VR systems embedded in neurorehabilitative devices such as treadmill (for lower limb recovery, [20]) or robotic arm systems [21].

Beyond clinical applications, an emerging line of research has begun to investigate whether combining tDCS during VR-based training (tDCS + VR) may modulate cognitive, affective, and behavioral processes in healthy or non-clinical populations. VR can provide immersive, controllable, and ecologically valid environments, whereas tDCS may transiently modulate cortical excitability in networks involved in attention, emotional regulation, or skill acquisition [22,23,24,25]. However, the available evidence remains sparse and methodologically heterogeneous. Importantly, most studies do not include the full set of control conditions required to determine whether VR and tDCS have independent, additive, or interactive effects. In particular, comparisons are often limited to active tDCS plus VR versus sham tDCS plus VR, which allows assessment of whether tDCS adds value during VR exposure but does not establish synergy between the two interventions. A systematic synthesis is therefore warranted not only to summarize the reported outcomes, but also to clarify what can and cannot be inferred from the current study designs.

## 2. Materials and Methods

### 2.1. Protocol

The protocol of this systematic review was registered on PROSPERO (CRD420251169010), following the Preferred Reporting Items for Systematic Reviews and Meta-Analyses (PRISMA) [26].

### 2.2. Main Outcomes

Given the increasing integration of immersive technologies and non-invasive brain stimulation in health promotion, training, and cognitive enhancement research, this review aimed to synthesize the outcomes reported in studies that combined tDCS with VR in non-clinical populations. The review addressed the following question: *Does active tDCS provide additional benefit when delivered during VR-based interventions or tasks in healthy/non-clinical participants?*

Primary outcomes included psychological and cognitive variables, such as anxiety, stress, affective responses, sustained and selective attention, inhibitory control, working memory, and motor learning. Secondary outcomes included acceptability, cybersickness, sensory immersion, presence, user engagement, cognitive workload, fatigue, neurophysiological indicators, and adverse effects or tolerability of tDCS. Because the included studies used heterogeneous designs and most did not include independent VR-only and tDCS-only arms, the synthesis focused on the direction and strength of evidence for an incremental effect of active tDCS during VR in non-clinical populations, rather than on claims of synergistic interaction.

### 2.3. PICO Model

The PICO framework was used to define the eligibility criteria and search strategy. The population comprised healthy or non-clinical participants. The intervention was simultaneous administration of active tDCS during a VR-based task or intervention. The primary comparator was sham tDCS delivered during the same VR exposure, or, in some studies, another VR-based control condition. Outcomes included quantitative cognitive, psychological, behavioral, motor, neurophysiological, tolerability, or presence-related measures.

Importantly, the PICO framework was interpreted in accordance with the inclusion criteria: studies were eligible only when tDCS and VR were delivered within the same experimental protocol. Therefore, this review does not provide a direct test of the independent effects of VR alone, tDCS alone, or their statistical interaction. Instead, the included literature mainly evaluates whether active tDCS produces additional effects when administered during VR.

### 2.4. Search Strategy

A systematic literature search was conducted in three electronic bibliographic databases (PubMed, Scopus, and IEEE Xplore) to identify studies published between January 2000 and September 2025 investigating the combined use of transcranial direct current stimulation (tDCS) and virtual reality (VR) in healthy or non-clinical populations. The final search was performed in September 2025. The search strategy was developed using combinations of free-text terms and controlled vocabulary where available (e.g., MeSH terms in PubMed). Across all databases, the core search concept combined terms related to non-invasive brain stimulation and immersive technologies using the expression (“tDCS” OR “transcranial direct current stimulation”) AND (“VR” OR “virtual reality” OR “immersive reality”). In PubMed, searches were performed within title/abstract fields and supplemented with MeSH terms when available. In Scopus, searches were conducted using the TITLE-ABS-KEY function, whereas in IEEE Xplore the same search terms were applied across all metadata fields. Database-specific adaptations were made to accommodate the indexing structure and search functionalities of each platform.

Eligibility criteria were defined a priori according to the review objectives. Studies were included if they: (a) involved healthy or non-clinical human participants; (b) investigated the concurrent application of tDCS and VR within the same experimental protocol; (c) were original empirical studies (e.g., randomized controlled trials, pilot studies, experimental studies, or case series); (d) were published in English; and (e) were published between January 2000 and September 2025.

Studies were excluded if they: (i) involved animal models; (ii) investigated VR-related technologies that did not include immersive or semi-immersive virtual reality environments (e.g., exergaming or serious games without VR); or (iii) included participants with diagnosed neurological, psychiatric, or medical disorders.

Title and abstract screening were independently performed by five reviewers (C.M., M.B., P.B., N.T., and A.A.). Potentially eligible studies underwent full-text assessment. Disagreements regarding study eligibility were resolved through discussion and consensus and, when necessary, by consultation with a sixth reviewer (A.C.). Data extraction was performed independently by the reviewers using pre-specified extraction criteria to minimize selection and reporting bias. To maximize retrieval of psychologically oriented studies, an additional search was conducted in the PsycINFO database using the same search terms and eligibility criteria. This supplementary search did not identify any additional studies meeting the inclusion criteria.

### 2.5. Synthesis of Evidence

**Figure 1:**
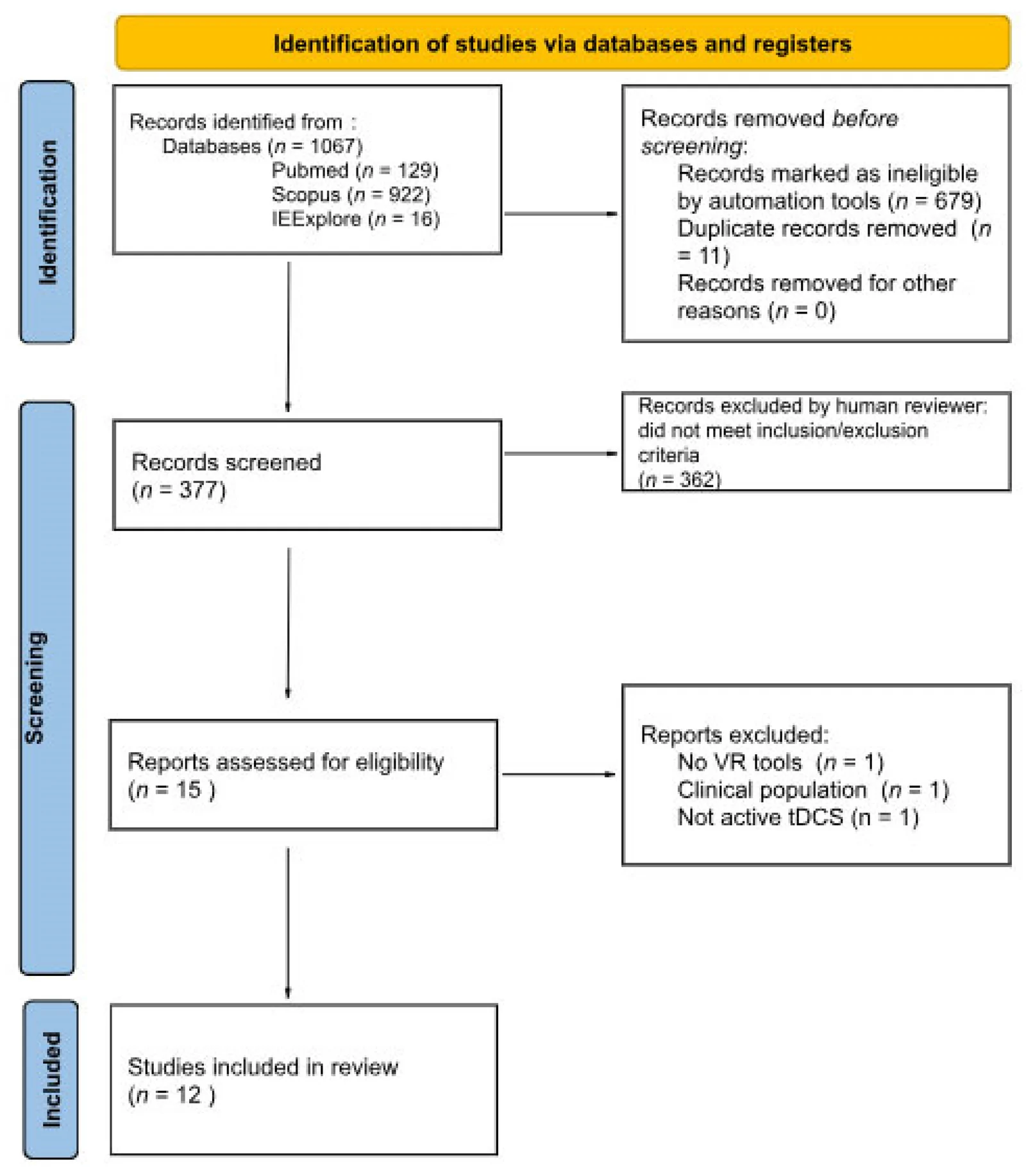
PRISMA flow diagram.

The study selection process followed PRISMA 2020 recommendations and is summarized in Figure 1. A total of 1067 records were identified through database searching. Prior to screening, automated database filters were applied to restrict results to studies published in English, conducted in humans, and classified as research articles. This procedure excluded 679 records. Subsequently, 11 duplicate records were identified and removed. The remaining 377 records underwent title and abstract screening. Of these, 362 studies were excluded because they did not meet the predefined eligibility criteria. Fifteen articles were retrieved for full-text assessment. Following full-text review, three studies were excluded because they did not meet the inclusion criteria (absence of VR intervention, absence of active tDCS intervention, or inclusion of clinical populations).

Ultimately, 12 studies met all eligibility criteria and were included in the qualitative synthesis. Given the substantial heterogeneity across studies in terms of design, participant characteristics, VR systems, stimulation protocols, outcome measures, and control conditions, a quantitative meta-analysis was not considered appropriate. Therefore, findings were synthesized narratively, with particular emphasis on study design, direction of effects, methodological quality, consistency of findings, and the extent to which the available evidence supports an incremental effect of active tDCS during VR exposure.

### 2.6. Quality Assessment and Risk of Bias in Randomized Control Trials (RCTs)

The methodological quality of the included randomized controlled trials (RCTs) was assessed using the revised Cochrane Risk of Bias tool for randomized trials (RoB 2) [27]. This tool evaluates five domains of potential bias: (i) bias arising from the randomization process, (ii) bias due to deviations from intended interventions, (iii) bias due to missing outcome data, (iv) bias in measurement of the outcome, and (v) bias in selection of the reported result. Each domain was judged as low risk, some concerns, or high risk according to the RoB 2 guidance, and an overall risk-of-bias judgment was assigned using the RoB 2 algorithm, whereby the overall judgment cannot be more favorable than the highest level of risk identified across domains.

**Figure 2:**
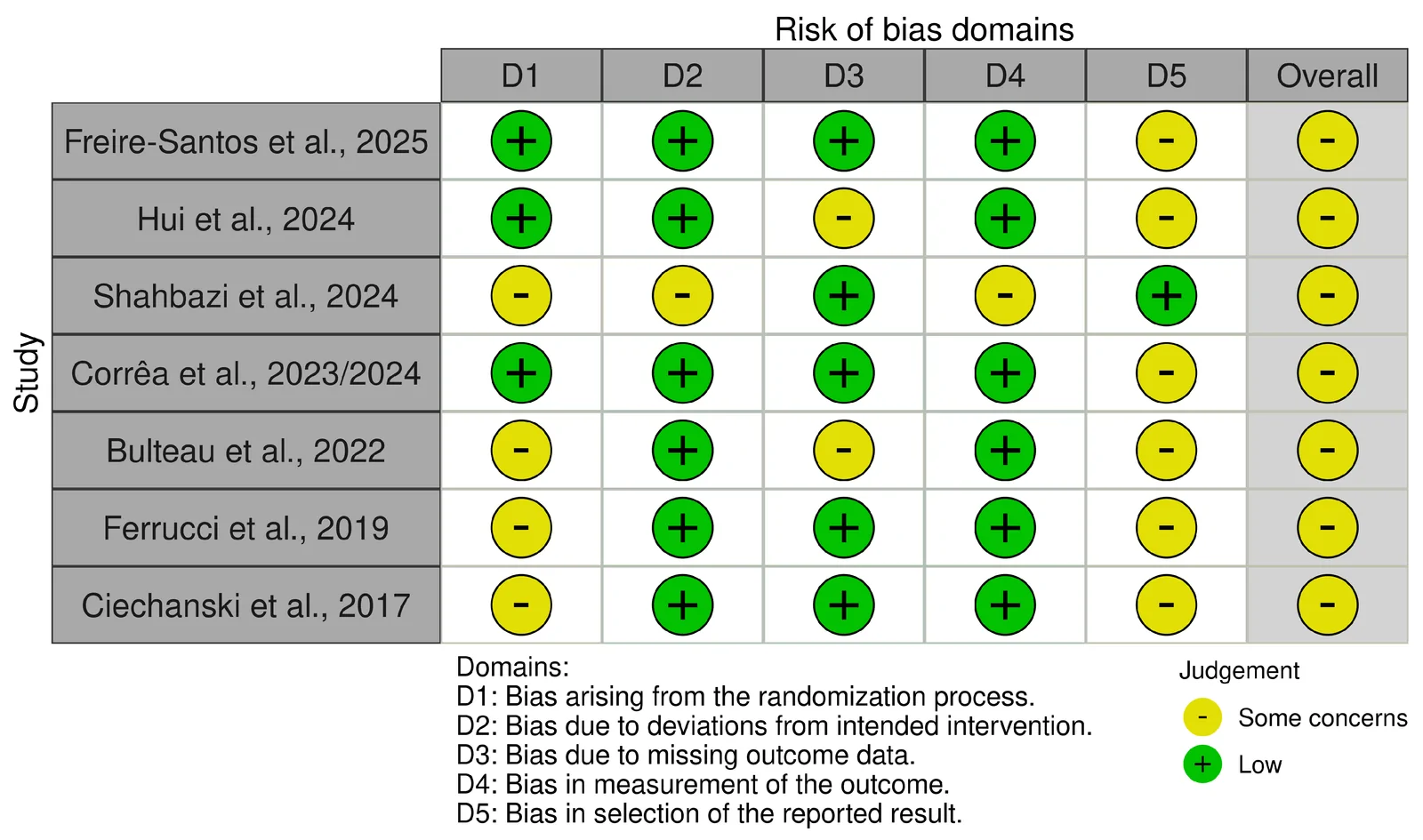
Risk-of-bias assessment of randomized controlled trials (RoB 2) [18,28,29,30,31,32,33].

**Figure 3:**
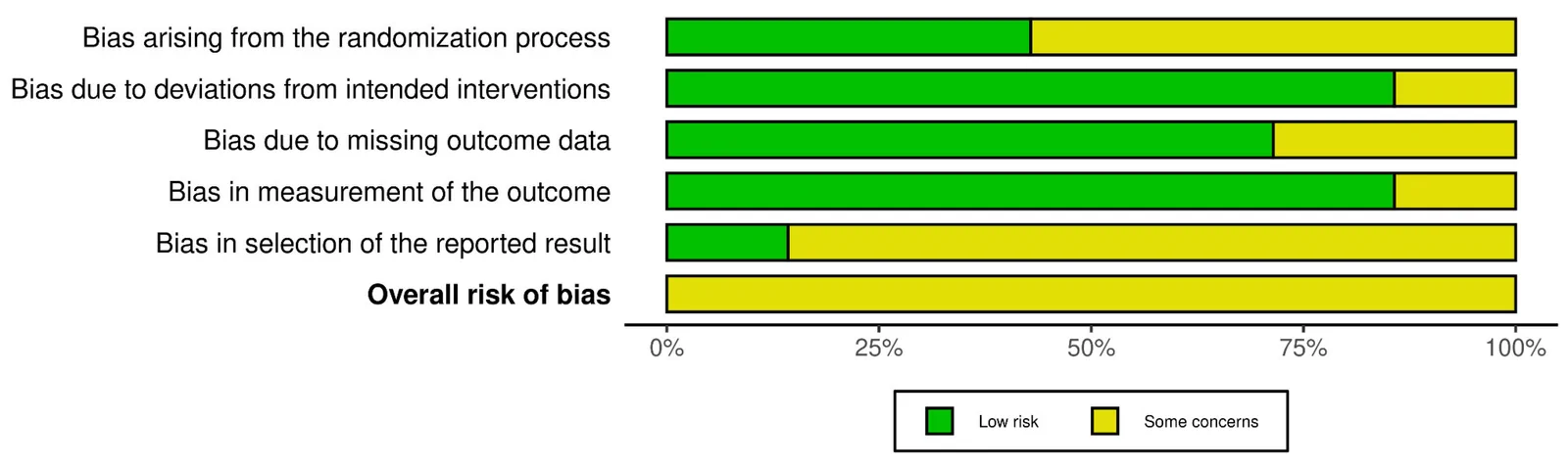
Summary of risk-of-bias assessment across randomized controlled trials (RoB 2).

Risk-of-bias assessments were performed independently by two reviewers (CM and MGM). Any discrepancies were resolved through discussion until consensus was reached. The detailed domain-level assessments and overall judgments are presented in Figure 2, whereas the distribution of risk-of-bias judgments across studies is summarized in Figure 3.

### 2.7. Risk of Bias of Experimental Neuromodulation Studies with Crossover or Laboratory-Based Designs (ROBINS-I)

Five studies were experimental neuromodulation studies characterized by crossover, laboratory-based, or proof-of-concept designs. These studies differed substantially from conventional clinical randomized controlled trials in terms of study design and reporting, particularly regarding the description of randomization procedures and allocation methods. Given this methodological heterogeneity and the limited information available to judge key aspects of the randomization process, these studies were evaluated separately using the Risk Of Bias In Non-randomized Studies of Interventions (ROBINS-I) tool [34]. The ROBINS-I tool assesses bias across seven domains: (i) bias due to confounding, (ii) bias in the selection of participants, (iii) bias in the classification of interventions, (iv) bias due to deviations from intended interventions, (v) bias due to missing data, (vi) bias in the measurement of outcomes, and (vii) bias in the selection of the reported result. Any disagreements in the risk-of-bias assessment were resolved through discussion until consensus was reached.

## 3. Results

The electronic search strategy conducted across three bibliographic databases retrieved 1067 studies. After screening titles and abstracts and removing duplicates, 15 studies remained for full-text review. Of these, three studies were excluded because they did not use VR or active tDCS or did not have a healthy population. Ultimately, 12 articles were included in this review (Table 1).

Before interpreting the findings reported in Table 1, it is important to clarify the inferential limits of the designs included in this review. Most studies compared active tDCS delivered during VR with sham tDCS delivered during the same VR exposure. Such designs can estimate whether active stimulation provides an additional benefit relative to sham stimulation during VR [18,19,20,21,22,23,24,25,26,27,28,29,30,31,32,33,34,38,39], but they cannot determine whether VR and tDCS have independent effects or whether their combination produces a true interaction. In addition, several studies included small experimental arms and assessed multiple outcomes or multiple group contrasts [28,29,30,31,32,38,39]. Where correction for multiple comparisons was absent or unclear, nominally significant or borderline findings should be interpreted cautiously [30,31,32,33,35,36,37,38,39]. Accordingly, the results below are summarized as evidence for possible incremental effects of active tDCS during VR rather than as evidence of synergistic efficacy.

### 3.1. Study and Sample Characteristics

The sample sizes of the 12 studies included in this analysis varied from 10 to 107 participants, with over 100 participants in only one study [28]. The participants’ ages ranged from 15 to 80 years old. Eight studies included healthy volunteers not divided by social/work classes (n = 397) [28,29,30,31,32,33,36,37,38,39], three studies included university students (n = 148) [18,19,20,21,22,23,24,25,26,27,28,29,30,31,32,33,34,35,36,37,38,39], and only one study included sedentary teenage girls (n = 36) [29]. Both male (n = 207) and female (n = 344) individuals were included.

Study designs were heterogeneous and included randomized controlled trials [18,19,20,21,22,23,24,25,26,27,28,29,30,31,32,33,34,38,39], repeated-measures designs [30,31,32,33,35,36,37,38,39], and mixed experimental designs [36,37].

### 3.2. Quality of Included Studies: Risk of Bias

Seven of the twelve included studies were evaluated using the revised Cochrane Risk of Bias tool for randomized trials (RoB 2) [27]. Overall, all randomized controlled trials were judged to present some concerns according to the RoB 2 algorithm, although the domains contributing to these judgments differed across studies (Figure 2). Concerns regarding Domain 1 (bias arising from the randomization process) were identified in four of the seven studies [29,30,31,32,33,38,39], primarily because the methods used for sequence generation and/or allocation concealment were insufficiently described. In contrast, Freire-Santos et al. [28], Hui et al. [18], and Corrêa et al. [30] were judged to be at low risk of bias for this domain. For Domain 2 (bias due to deviations from intended interventions), six studies were judged to be at low risk of bias, whereas Shahbazi et al. [29] was judged to present some concerns because of limited reporting regarding masking procedures and the inclusion of a no-intervention control group. Regarding Domain 3 (bias due to missing outcome data), five studies were judged to be at low risk of bias, whereas Hui et al. [18] and Bulteau et al. [31] were judged to present some concerns because of incomplete reporting regarding missing outcome data and their management. For Domain 4 (bias in measurement of the outcome), six studies were judged to be at low risk of bias, whereas Shahbazi et al. [29] was judged to present some concerns because blinding of outcome assessors was not clearly reported. The domain most frequently affected was Domain 5 (bias in selection of the reported result). Six of the seven randomized controlled trials were judged to present some concerns because insufficient information was available to determine whether the reported analyses had been pre-specified, precluding the exclusion of selective reporting bias. In contrast, Shahbazi et al. [29] was judged to be at low risk of bias in this domain because the reported outcomes were consistent with the prospectively registered trial protocol.

Overall, the randomized controlled trials demonstrated acceptable methodological quality. Although all studies were judged to present some concerns according to the RoB 2 algorithm, these judgments were generally driven by isolated methodological limitations rather than pervasive risks of bias. The most common concerns related to the reporting of the randomization process (Domain 1) and the availability of pre-specified analyses (Domain 5), whereas concerns regarding missing outcome data (Domain 3) and outcome measurement (Domain 4) were limited to a small number of studies.

**Figure 4:**
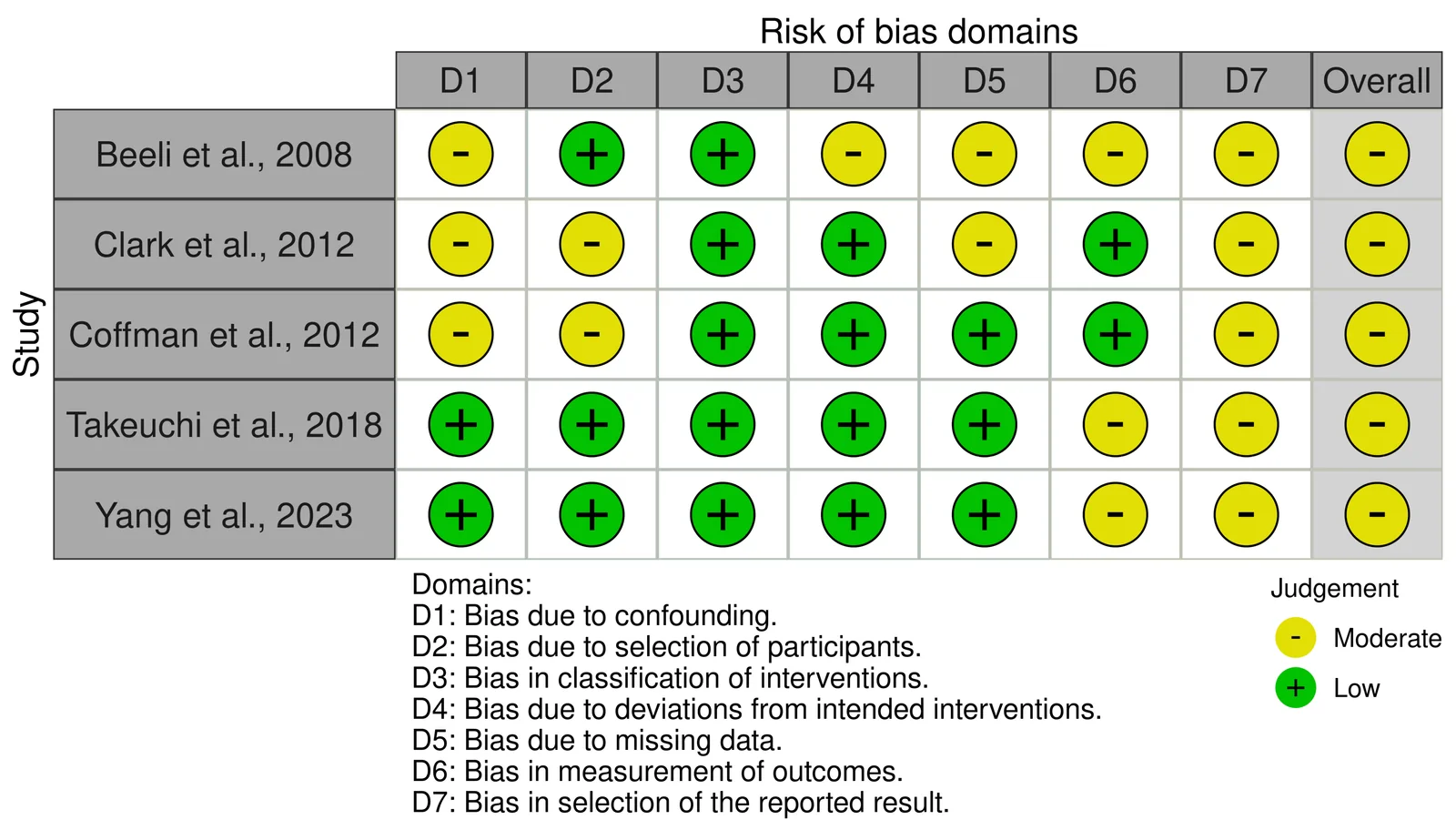
**Domain-level ROBINS-I risk-of-bias judgments for each experimental neuromodulation study** [35,36,37,38,39].

**Figure 5:**
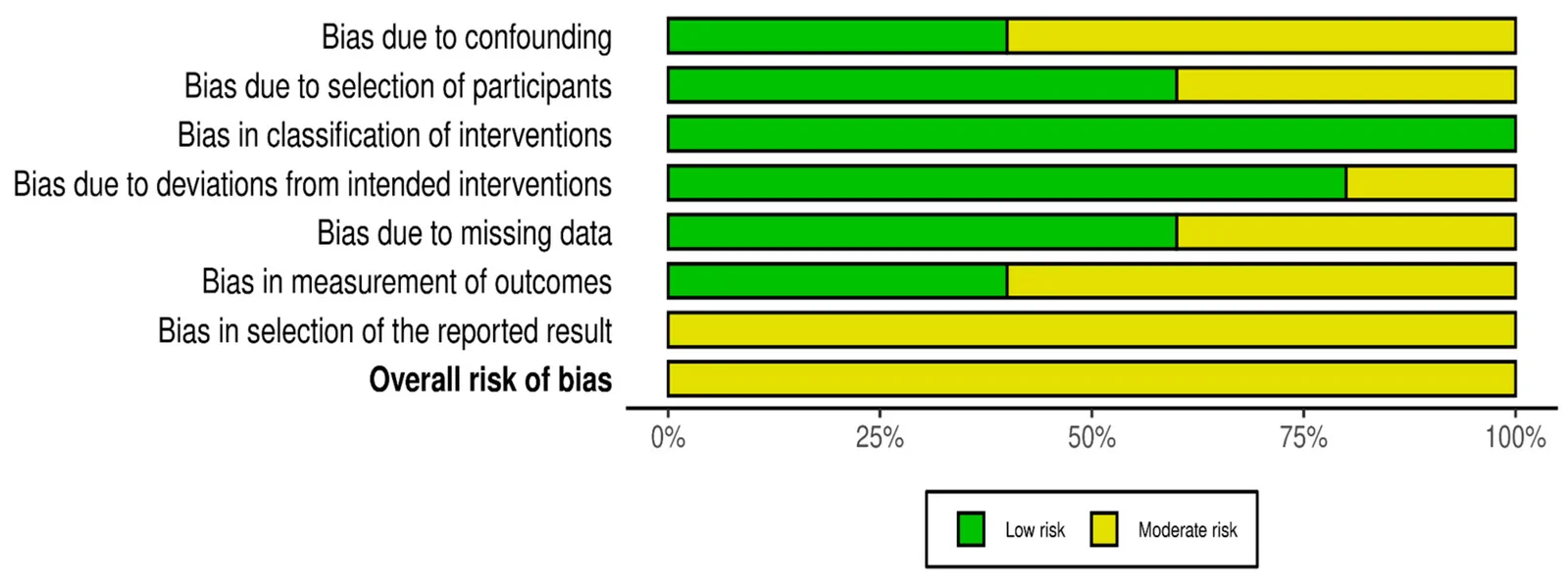
**Summary of risk-of-bias judgments across ROBINS-I domains**.

The ROBINS-I assessment indicated that all five experimental neuromodulation studies were classified as having an overall moderate risk of bias (Figure 4 and Figure 5). Low risk of bias was consistently observed for the classification of interventions (D3), while most studies were also judged to have a low risk of bias for participant selection (D2), deviations from intended interventions (D4), and missing data (D5). Moderate concerns were more frequently identified for bias due to confounding (D1), measurement of outcomes (D6), and particularly the selection of the reported result (D7), primarily owing to limited reporting of pre-specified analytical plans and the inclusion of subjective outcome measures in several studies. Moderate concerns regarding participant selection and missing data were identified in a small number of studies because of post-intervention participant exclusions or incomplete physiological recordings. Overall, all five experimental neuromodulation studies were classified as having a moderate risk of bias according to the ROBINS-I framework. These overall judgments were primarily driven by moderate concerns related to confounding, outcome measurement, and the selection of the reported result, whereas the remaining domains were generally judged to be at low risk of bias.

### 3.3. Methodological Approaches to Implementing VR with tDCS

In every study included in this review, the methodological approaches used were the combination of VR and tDCS. The combined use of these two technologies resulted in significant variation among investigations; in fact, numerous tools and approaches have been used to provide VR, and different areas of the brain have been the focus of different investigations.

The VR systems used in the twelve studies included in this review are immersive or semi-immersive. The immersive VR systems are delivered by 3D headset: Oculus Rift [28,29,30,31,32,38], HTC Vive Pro [31,32,39], Meta quest 2 [18], and Oculus quest [38]. The semi-immersive VR systems are delivered by computer [35], Xbox360 [29], projector [30,31,32,33,36,37,39] and stereovision system [33].

Since the EEG 10–20 approach was used in all studies to locate the cortical areas of interest, the tDCS systems were uniform in their design. However, as Figure 4 and Figure 5 show, there were significant differences between studies in terms of both the stimulation sites and the applied intensities.

Twelve studies used anodal stimulation to target different parts of the brain in relation to the active experimental groups: Clark et al. [36] and Coffman et al. [37] at the IFC, Beeli et al. [35], Corrêa et al. [30] and Freire-Santos et al. [28] at the dlPFC, Bulteau et al. [31] at the vmPFC, Ferrucci et al. [32] at the cerebellum, Ciechanski et al. [33] at the M1, Hui et al. [18] at the mPFC, Shahbazi et al. [29] at the M1 and dlPFC, Takeuchi et al. [39] at the TPJ, and Yang et al. [38] at the rVLPFC. Cathodal stimulation in the active group was applied in two studies: Beeli et al. [35] at the r-dlPFC and Takeuchi et al. [39] at the TPJ. The experimental group in four investigations [28,29,30,31,32,33,35,36,37,38,39] was designed to include a sham tDCS condition.

For the control group, three studies included a no-tDCS condition [28,29,30,38], while six studies used sham-tDCS [18,19,20,21,22,23,24,25,26,27,28,29,30,31,32,33,34,38,39].

Of the 12 studies in this review, four used cognitive tasks in VR with anodal stimulation over frontal regions (PFC or IFC) to modulate attentional and inhibitory capacities [30,31,32,33,36,37,38,39]. Only Ferrucci et al. [32], which aimed to modulate spatial abilities, stimulated the cerebellum. Anodal tDCS over the PFC was combined with VRET protocols in two studies [18,19,20,21,22,23,24,25,26,27,28,29,30,31,34,38] to modulate psychological variables (stress, depression, anxiety), while VR games were used in two other studies [29,30,31,32,33,35,36,37,38,39] to modulate motor and balance-related variables. More specific techniques were used in the other studies. For instance, Freire-Santos et al. [28] employed VR-based mindfulness, and Ciechanski et al. (2017) used a VR simulation program in conjunction with excitatory stimulation over M1 to acquire surgical skills.

### 3.4. Outcome

**Figure 6:**
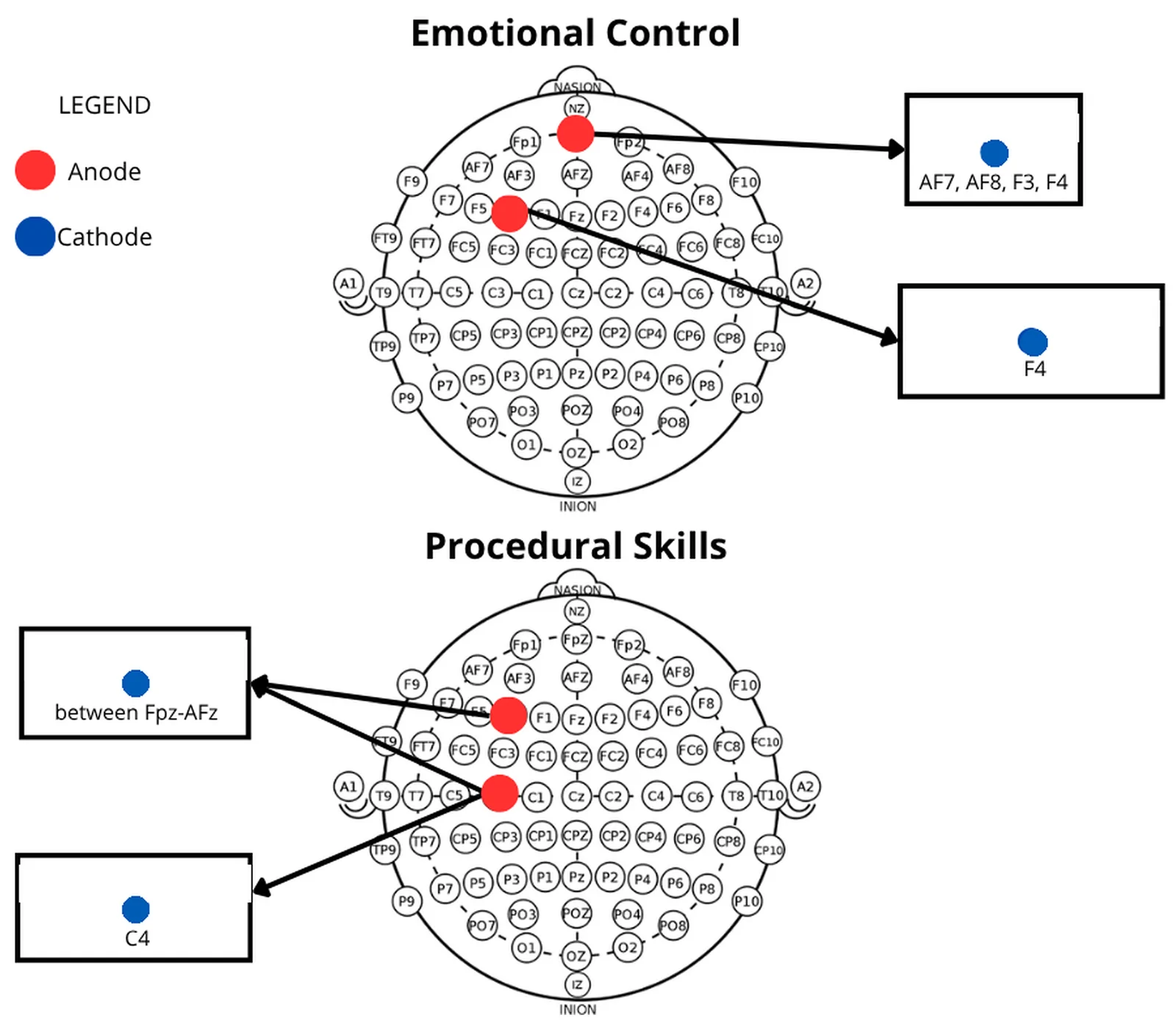
**Main stimulation targets associated with significant outcomes.** Summary of the stimulation montages from RCT in which active tDCS + VR produced significant behavioral improvements compared with sham tDCS, VR-only, or no-treatment control conditions. Stimulation protocols are grouped by outcome domain (emotional control and procedural skills). The figure illustrates the location of the anodal electrode (red circles) according to the International 10–20 EEG System, while the corresponding cathodal electrode (blue circles) and the associated study are indicated for each montage. For the “Emotional Control” applications, anodal localization in FpZ refers to the paper of Hui et al., 2024 [18], while the localization in F3 refers to the paper of Freire-Santos et al., (2025) [28]. For the “Procedural Skills” applications, anodal localization in F3 refers to the paper of Shahbazi et al., 2024 [29], while the localization in C3 refers to the paper of Ciechanski et al., 2017 [33]. The figure reports only stimulation protocols supported by significant between-group comparisons. Within-subject or repeated-measures findings are described in the text but are not represented in the figure.

**Figure 7:**
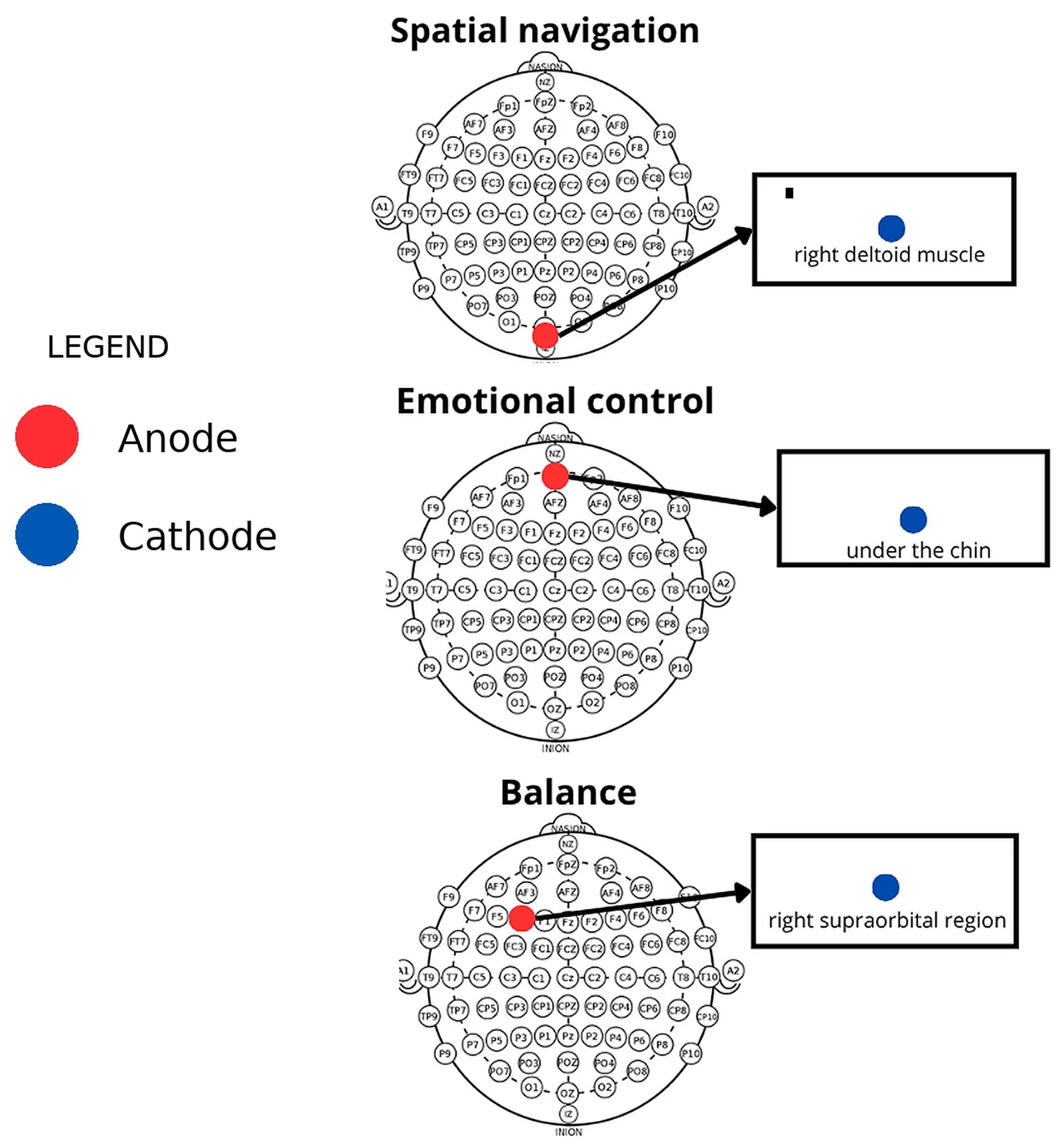
**Brain stimulation targets associated with null effects in RCT.** Summary of the tDCS electrode montages that did not yield significant between-group behavioral effects in studies comparing active tDCS + VR with sham tDCS, VR-only, or no-treatment control conditions. Stimulation protocols are grouped according to the outcome domain (spatial navigation, emotional control, and cybersickness). The head maps illustrate the location of the anodal electrode according to the International 10–20 EEG System (red circles), while the corresponding cathodal electrode position (blue circles) and the related study are reported alongside each montage. For the “Spatial navigation” application, anodal localization in OZ refers to the paper of Ferrucci et al., 2019 [32], For the “Emotional Control” applications, anodal localization in FpZ refers to the paper of Bulteau et al., 2022 [31]. For the “Balance” applications, anodal localization in F3 refers to the paper of Correa et al., 2023 [30]. Only stimulation protocols for which no significant between-group differences were observed are presented.

As shown in the last column of Table 1, the application of the combined tDCS + VR approach yields different positive or null outcome improvements. Moreover, the tDCS application on brain regions is also characterized by spatial heterogeneity. For this reason, Figure 6 summarizes the main significant positive findings in RCT studies, categorized as tDCS stimulation combined with VR training for improving emotional control (anxiety, impulsivity, and skin conductance response) or procedural skills (motor coordination, neurosurgical skills, motor balance), over the brain stimulated regions. Otherwise, Figure 7 summarizes the main null effects.

However, caution is required because some studies reported improvements in both active and sham/control VR conditions, suggesting that VR training or exposure itself may account for part of the observed benefit. In addition, studies differed substantially in stimulation targets, VR platforms, task demands, sample characteristics, and statistical approaches.

#### 3.4.1. Cognitive Outcomes (Attention, Vigilance, Signal Detection, Learning)

Several randomized controlled trials examined cognitive outcomes, including sustained attention, inhibitory control, spatial navigation, and procedural skill acquisition. Overall, the evidence was mixed. Freire-Santos et al. [28] investigated the effects of active versus sham tDCS combined with virtual reality-based mindfulness on attention and inhibitory control. No significant between-group differences were observed on the Emotional Stroop Task or the Sustained Attention to Response Task, indicating that active stimulation did not confer measurable advantages over the corresponding sham conditions on these cognitive outcomes. Similarly, Ferrucci et al. [32] found no significant differences between active cerebellar tDCS and sham stimulation during a virtual spatial navigation paradigm, suggesting that active stimulation did not enhance performance in spatial learning or navigation beyond the effects associated with the VR task itself. Taken together, the available RCT evidence provides limited support for a generalized cognitive-enhancing effect of tDCS during VR exposure. Most sham-controlled studies evaluating attention, inhibitory control, or spatial cognition failed to demonstrate significant between-group advantages for active stimulation. Consequently, current RCT findings suggest that any cognitive benefits of tDCS during VR are likely to be task-specific rather than broad or consistently replicable.

#### 3.4.2. Emotional and Psychological Outcomes (Anxiety, Emotional Regulation, Discomfort, Physiological Arousal)

Four studies examined psychological outcomes, including anxiety, stress, depressive symptoms, discomfort, or physiological indices of emotional reactivity [18,19,20,21,22,23,24,25,26,27,28,29,30,31,32,33,34,35,36,37,38,39]. The most consistent evidence for an additional effect of active tDCS during VR emerged in anxiety-related or emotional-regulation contexts. In Freire-Santos et al. [28], participants receiving the combined VR-focused mindfulness plus active tDCS intervention differed significantly from those receiving VR mind-wandering plus sham tDCS (*p* = 0.014), yielding a significant main effect of group (*F*(3,66) = 4.07, *p* = 0.010, η^2^ = 0.156) on the non-specific skin conductance response (nsSCR). These authors concluded that tDCS + VR induces a potential modulatory effect on autonomic arousal rather than on cognitive performance outcomes. Hui et al. [18] reported greater reductions in anxiety-related measures and discomfort in the active tDCS + VRET group compared with sham tDCS + VRET] (Figure 6). In contrast, another study found no clear additional effect of active stimulation beyond VR exposure on the anxiety level [31] (Figure 7). These mixed findings suggest that VR exposure itself may produce meaningful psychological change, while the incremental contribution of tDCS may depend on stimulation target, symptom severity, task context, and outcome sensitivity.

#### 3.4.3. Motor and Skill-Related Outcomes

Several studies examined outcomes related to motor performance, motor coordination, balance, and skill acquisition. Evidence across these domains was heterogeneous. In some studies, active tDCS delivered during VR was associated with superior performance compared with sham stimulation delivered during the same VR intervention, suggesting a possible incremental contribution of active stimulation (Figure 6). For example, Ciechanski et al. [33] reported significantly greater acquisition of neurosurgical skills following active tDCS combined with VR simulation training compared with sham stimulation, whereas Shahbazi et al. [29] did not demonstrate an immediate advantage of adding tDCS to VR training, as the two VR groups did not differ significantly at post-intervention. However, the added value of tDCS emerged during the retention phase. Participants who received VR combined with active dual-site tDCS (targeting M1 and DLPFC) showed significantly better retention of motor learning than those who received VR combined with sham stimulation, both in eye–hand coordination and bimanual coordination. These findings suggest that active tDCS may enhance the consolidation and long-term retention of motor skills acquired through VR training, rather than producing stronger immediate performance gains. This evidence was not confirmed by Corrêa et al. [30] who found no significant differences between active and sham stimulation conditions when combined with VR-based training for postural balance.

Overall, the available evidence suggests that combined tDCS + VR protocols are feasible and may provide additional benefits in selected motor and skill-learning contexts.

#### 3.4.4. Evidence from Non-Randomized, Repeated-Measures, and Mixed Experimental Studies

In addition to randomized controlled trials, several included studies employed repeated-measures, crossover, or mixed experimental designs. Although these investigations provide potentially valuable information regarding the modulation of VR-related outcomes by active tDCS, their results should be interpreted with caution because they generally offer a lower level of inferential certainty than sham-controlled parallel-group RCTs. Overall, these studies suggested possible benefits of active stimulation on attention, signal detection, learning, cybersickness-related symptoms, and behavioral control. Yang et al. [38] reported improvements in perceived attention, task accuracy, and reaction time during active HD-tDCS combined with VR compared with the sham condition. Similarly, Clark et al. [36] and Coffman et al. [37] observed enhanced learning and target-detection performance during VR-based training following active frontal stimulation. Takeuchi et al. [39] found reductions in disorientation symptoms and postural instability during immersive VR exposure following active stimulation of the temporoparietal junction, while Beeli et al. [35] reported modulation of impulsive behavior during a VR-based paradigm. However, these findings should be considered exploratory rather than confirmatory. Most studies involved small samples, multiple outcome measures, repeated testing procedures, or several statistical contrasts, increasing susceptibility to practice effects, carry-over effects, expectancy-related influences, and selective outcome reporting. Furthermore, the absence of independent VR-only and tDCS-only control conditions prevents determination of whether the observed changes were attributable to active stimulation, VR exposure, task repetition, or a combination of these factors. Consequently, the available non-RCT evidence should be regarded as hypothesis-generating and supportive of future investigation, rather than as definitive evidence that active tDCS enhances VR-related outcomes.

## 4. Discussion

The combination of PTs and non-invasive neuromodulation tools is a new field of study that has already been effective in neurological and psychiatric populations [16,40], but it is in its relative infancy in non-clinical populations. The rationale for combining VR and tDCS is based on evidence suggesting that both interventions can independently influence cognitive, emotional, and behavioral processes through partially distinct mechanisms. In several neurological and psychiatric disorders this new combined approach would seem to be effective in reducing several symptoms (pain, binge eating disorder, anxiety disorders, etc.) [16,17]. In non-clinical populations, we evaluated if this approach can be applied to stimulate cognitive resources, boosting emotional control and general skills. What clearly emerged from this systematic review was that active tDCS may provide additional benefits during selected VR-based interventions or tasks, particularly in domains related to emotional regulation, physiological arousal, and skill acquisition. However, the findings must be interpreted with caution. Most included studies compared active tDCS plus VR with sham tDCS plus VR, meaning that they primarily tested whether tDCS adds value during VR exposure. They did not test whether VR and tDCS exert independent effects, nor whether their combination produces a true synergistic interaction. Therefore, the present evidence supports preliminary claims of possible incremental or augmentative effects, but not strong claims of synergy.

A further consideration is that several studies reported improvements in both active and sham/control VR conditions. In such cases, the findings may reflect the effects of VR training, task repetition, exposure, expectancy, or non-specific intervention factors rather than a specific contribution of active tDCS. This does not mean that the reported effects are invalid; rather, it means that the mechanistic interpretation should remain conservative. The current literature demonstrates feasibility and suggests promising domains for future research, but stronger experimental designs are needed before definitive conclusions can be drawn about combined efficacy. Importantly, the most robust evidence currently available derives from a limited number of randomized controlled studies, whereas several positive findings originate from small non-randomized or exploratory investigations. Consequently, the latter should be considered hypothesis-generating rather than confirmatory evidence.

### 4.1. Evidence for Possible Incremental Effects of tDCS During VR Stimulation

A rationale for combining VR and tDCS derives from evidence suggesting that both approaches can independently enhance cognitive performance in healthy individuals. On the one hand, immersive VR interventions have been associated with improvements in attentional engagement and sustained attention [41,42]. On the other hand, tDCS has been shown to modulate attention-related cortical networks, improving executive and attentional functions [43,44]. These observations provide the theoretical basis for combining both approaches within the same intervention. Several studies included in this review reported favorable outcomes when active tDCS was delivered concurrently with VR-based interventions. In the cognitive domain, improvements in attention-related performance, signal detection, and learning were observed in studies by Yang et al. [38], Coffman et al. [37], and Clark et al. [36]. However, these findings should be interpreted cautiously because the corresponding study designs generally compared active tDCS + VR with sham tDCS + VR and therefore assessed whether active stimulation provided an additional benefit during VR exposure rather than whether the combined intervention was superior to either modality alone.

A similar rationale applies to emotional regulation and inhibitory control. Previous research has shown that both VR-based interventions and tDCS can independently influence emotional and behavioral regulation in healthy individuals. Immersive VR environments, particularly exposure-based and interactive protocols, have been associated with improvements in emotional processing, anxiety and stress reduction [45,46]. Likewise, tDCS has been shown to modulate prefrontal networks involved in cognitive control, decision-making, and emotion regulation, leading to improvements in impulsivity- and inhibition-related processes [47,48,49]. These observations provide the theoretical basis for combining the two approaches within the same intervention. Within the studies included in this review, evidence for emotional outcomes was observed primarily in anxiety- and arousal-related measures. Hui et al. [18] reported greater reductions in anxiety and discomfort following active tDCS combined with VRET compared with sham stimulation delivered during the same VR protocol. Similarly, Freire-Santos et al. [28] observed a greater reduction in physiological arousal, indexed by non-specific skin conductance responses, when active tDCS was combined with VR-based mindfulness. In contrast, Bulteau et al. [31] did not detect additional benefits of active stimulation beyond VR exposure alone. Taken together, these findings suggest that active tDCS may enhance selected emotional-regulation outcomes during VR-based interventions, although the available evidence remains insufficient to establish whether such effects reflect true interaction effects between the two modalities or simply an incremental contribution of active stimulation.

Regarding motor function, some research indicates that VR improves EHC and eye–body coordination in kids and teenagers [50,51]. According to recent research, anodal tDCS across the M1 region accelerates the acquisition of new skills [52]. Combining tDCS + VR, Ciechanski et al. [33] demonstrated superior neurosurgical skill acquisition following active tDCS combined with VR simulation, whereas Shahbazi et al. [29] reported improvements in both active and sham stimulation groups relative to no-treatment controls. In the latter case, the comparable improvements observed in the active and sham VR conditions suggest that VR-based training itself may have been a major contributor to the observed behavioral gains. Consequently, these findings support the feasibility and potential utility of combined protocols but provide only limited evidence regarding the specific contribution of active tDCS.

Overall, the current literature suggests that active tDCS may, under certain conditions, incrementally enhance selected VR-based outcomes. However, because no included study employed a fully factorial design capable of disentangling independent, additive, and interaction effects, conclusions should remain restricted to possible augmentation effects of active tDCS during VR rather than claims of synergy between the two interventions.

### 4.2. Interpreting Sham-Controlled VR Studies

Notwithstanding encouraging results, evidence in the current literature also showed that sham stimulation was adequate to produce the desired effect (see Figure 7). Generally, sham-controlled tDCS studies should also include a no-stimulation/no-device-exposure arm in order for interpretation as placebo responses [53]. Accordingly, most of the included studies used sham stimulation for methodological purposes and not for investigating psychological processes underlying expectancy-driven placebo effects, which can partially account for the observed behavioral changes.

Bulteau et al. [31] examined the lack of impact on psychological outcomes and found that active tDCS administered to the vmPFC compared to sham tDCS did not improve the therapeutic effects of VR on anxiety or discomfort associated with height. The tDCS stimulation may not have been strong enough or specifically focused to regulate the cortical regions linked to acrophobia (such as the insula and parietal regions involved in visuo-vestibular processing). Furthermore, as baseline symptoms were relatively low, the selection of healthy individuals with just mild types of visual intolerance may have limited the detection of any clinically significant effect. Additionally, the authors hypothesized that VR itself would have a ceiling effect: immersive exposure alone appeared to be adequate in lowering anxiety and boosting familiarity with heights, making it challenging to identify any further advantages from tDCS. In conclusion, the study did not show a clear benefit of brain stimulation over VR exposure alone, despite establishing the viability and safety of combining VR and tDCS. Ferrucci et al. [32] found multiple possible explanations for the lack of difference between the anodal stimulation of the cerebellum to sham stimulation combined with a spatial navigation in VR. Since allocentric/egocentric processing and spatial navigation involve more complex neural circuits (like the hippocampus) that are not directly stimulated, the cerebellum may not be the best target for modulating the retrieval phase of spatial mapping in VR. Additionally, cerebellar tDCS is still poorly characterized in terms of stimulation parameters (electrode montage, intensity, and duration). Additionally, the chance to alter spatial learning in real time may have been diminished because stimulation was given before the retrieval phase rather than during active navigation.

According to Correa et al. [30], tDCS active or sham with VGT did not improve postural balance in older women more than video game training alone. The primary cause of this result, according to the authors, was a ceiling effect: the video game program on its own showed adequate efficacy in improving balance, which decreased the possibility of finding additional effects from the addition of tDCS. Additionally, the stimulation may not have been appropriately directed towards the brain regions more directly involved in postural control because it focused on the DLPFC. The sample’s limited clinical vulnerability is another potential contributing factor. The participants were older women who lived in the community rather than people with severe motor impairments, so their margin for improvement was constrained. In conclusion, the combination of these elements seems to be the cause of the combined intervention’s failure.

### 4.3. Limitations

Several limitations of the current literature should be emphasized. First, none of the included studies used a full factorial design crossing VR and tDCS conditions. A design including active tDCS + VR, active tDCS without VR, sham tDCS + VR, and sham/no stimulation without VR would be required to test whether the two interventions interact synergistically. As a result, the available studies cannot determine whether the combined effect exceeds the sum of the two interventions administered separately. The evidence therefore supports only cautious conclusions regarding possible incremental effects of active tDCS during VR exposure. Second, many studies used small experimental arms. This limits statistical power, reduces precision around effect estimates, and increases the risk that only large or unstable effects are detected. Small samples also make it difficult to evaluate moderators such as age, baseline performance, VR experience, stimulation sensitivity, or psychological symptom severity. Third, several studies examined multiple outcomes, time points, or group contrasts, while correction for multiple comparisons was not always clearly reported. This is particularly relevant for borderline significant findings, which should be interpreted as preliminary unless supported by pre-specified hypotheses, correction procedures, or replication. All this evidence, together with the fact that most included studies did not report standardized effect sizes, confidence intervals, or sufficient statistical information to calculate them, also limit a formal meta-analysis. Fourth, the included studies were heterogeneous in VR systems, stimulation targets, electrode configurations, stimulation parameters, outcome measures, and sham/control designs. Finally, an additional limitation concerns the heterogeneity of participant age. Although most studies enrolled adults, one included study [29] recruited adolescents aged 15–18 years, whereas others included older adults up to 80 years of age. Consequently, the present synthesis combines findings across distinct developmental stages. Because physiological responses, tolerability, and optimal stimulation parameters for tDCS may differ across age groups, the current evidence should not be interpreted as supporting identical efficacy or safety profiles across adolescents, younger adults, and older adults.

This heterogeneity prevents strong pooled conclusions and limits mechanistic interpretation. Future research should adopt more standardized reporting of stimulation parameters, VR characteristics, adverse effects, tolerability, presence, and task engagement.

## 5. Conclusions

The available evidence suggests that active tDCS may provide additional benefits during selected VR-based interventions in healthy and non-clinical populations, although the effects appear to be domain-specific rather than generalized. The strongest evidence emerged from randomized controlled trials, where active stimulation combined with VR was associated with improvements in emotional regulation and physiological arousal (e.g., reduced anxiety, discomfort, and autonomic reactivity) as well as procedural and motor skill learning, including neurosurgical skill acquisition and retention of motor coordination. In contrast, RCT findings did not consistently support additional benefits for broader cognitive outcomes, spatial navigation, balance training, or all anxiety-related VR paradigms.

Overall, the current literature supports the feasibility of combining tDCS and VR and indicates that active stimulation may incrementally augment selected VR-based outcomes under specific conditions. Nevertheless, the available evidence does not support strong claims of synergistic interaction between the two techniques. Most studies compared active versus sham stimulation during the same VR exposure and therefore assessed whether tDCS added value during VR rather than whether the combined intervention was superior to either modality alone. Future adequately powered, pre-registered factorial trials are needed to determine whether tDCS and VR exert independent, additive, or truly interactive effects.

## Figures and Tables

**Table 1:** Characteristics of studies applying VR systems and tDCS.

Author(s)	Population	Aims	Study Design	Intervention	VR-System	Tdcs Setup	Outcome Measures	Main Results
**Beeli et al.,** (2008) [35]	Sample size: 35; Population: university students; Age: *M* = 20.9, *SD* = 3.7; Sex: 17 females, 18 males.	Effects of tDCS-related prefrontal modulation on autonomic responses and impulsivity.	*Repeated measurements design* •Experimental 1 phase: anodal tDCS + VR •Experimental 2 phase: cathodal tDCS + VR •Control phase: sham tDCS + VR	**Anodal/Cathodal tDCS + VR:** One 5.5 min tDCS session, followed by Go/No-go task and a virtual roller-coaster scenario **Sham tDCS + VR:** One tDCS session, followed by the same Go/No-go task and virtual roller-coaster scenario. The three conditions were separated by a 3.5 min break.	Computer screen	•Region: right dlPFC •Position: In experimental 1 anode electrode on FC3 and cathode electrode on the ipsilateral mastoid. In experimental 2, they were switched. For control, it was switched off. •Application: Lasted 5.5 min at a constant current intensity of 1.5 mA. •Electrode dimensions/area (cm^2^): Saline sponge electrode:/35.	•EDA: SCR and SCL •EMG •Go-No-go task •MEC-SPQ •SAM	**Within results:** **↓** Reduced impulsive behavior control in experimental 2 intervention with respect to experimental 1 and control interventions (*F*(2,68) = 3.653; *p* = 0.03).
**Bulteau et al.,** (2022) [31]	Sample size: 25; Population: healthy participants; Age: *M* = 37; Sex: 18 females, 7 males.	Feasibility of combining tDCS with fully immersive VRET to reduce height anxiety.	*RCT—Two groups* • Experimental: anodal tDCS + VRET (*n* = 11) •Control: sham tDCS + VRET (*n* = 14)	Two 20 min sessions VRET concurrently active or sham tDCS.	HTC VIVE CV1 (Oculus VR, Menlo Park, CA, USA)	•Region: vmPFC •Position: Anode at FpZ, cathode under the chin. •Application: Experimental 1 mA anodal stimulation for 20 min; control 30 s of stimulation ramp-on and ramp-up. •Electrode dimensions/area (cm^2^): Saline rectangular sponge:/25.	•AQ •ATHQ •HIQ •vHIQ •STAI-Y •CGI •SUD •IPQ •SSQ	**Between-group results:** No significant effect.
**Ciechanski et al.,** (2017) [33]	Sample size: 22; Population: medical students; Age: *M* = 25.2; Sex: 16 females, 6 males.	Effects of tDCS on VR-based neurosurgical skill acquisition.	*RCT—double blind* •Experimental: anodal tDCS + VR (*n* = 11) •Control: sham tDCS + VR (*n* = 11)	One 24 min session of VR tumor-resection training with concurrent tDCS.	NeuroTouch Neurosurgical Simulator (developed by the National Research Council Canada)	•Region: M1 •Position: Anode C3, cathode C4. •Application: Experimental at 1 mA for 20 min; control at 1 mA for 60 s. •Electrode dimensions/area (cm^2^): Saline sponge electrode:/25.	•Percentage of tumor resected •Volume of healthy brain resected • Time of excessive forces	**Between-group results:** **↑** Improved resection efficiency in experimental respect to control groups (F1 = 12.863; *p* = 0.006).
**Clark et al.,** (2012) [36]	Sample size: 83; Population: healthy participants; Age: *M* = 24.1; Sex: 30 females, 53 males.	Cognitive mechanisms underlying anodal tDCS-enhanced performance in a VR target-detection task.	*Four-arm mixed design* •Experiment 1: Experimental 1 Group: anodal tDCS + VR (*n* = 13) Experimental 2 Group: sham tDCS + VR (*n* = 14) •Experiment 2: Experimental 3 Group: anodal tDCS + VR (*n* = 13) Experimental 4 Group: sham tDCS + VR (*n* = 23) •Experiment 3: Experimental 5 Group: sham tDCS +VR (*n* = 8) •Experiment 4: Experimental 6 Group: anodal tDCS + VR (*n* = 12)	One 1 h VR session with tDCS starting 5 min before training.	JVC-DLA Multimedia projector (ModelDLA-SX200-NLG)(JVC Kenwood Corporation, Yokohama, Japan)	•Region: Right IFC; right parietal cortex. •Position: Experiment 1, 2, 3 the anode was placed on F10, cathode at contralateral arm; experiment 4 the anode was placed on P4. •Application: 30 min, experimental 2 and experimental 4 at 0.1 mA; experimental 1, 3 and 6 at 2.0 mA; experimental 5 at 0.6 mA. •Electrode dimensions/area (cm^2^): Saline sponge electrode/11.	•Testing stimuli: images containing objects •Self-reported skill sensation	**Between-group results:** **↑** Improved learning during training (*F*(1,61) = 13.16, *p* = 0.0006), immediately after training (*F*(1,35) = 12.23, *p* = 0.001), and at 1 h follow-up (*F*(1,35) = 11.09, *p* = 0.002) in experimental 1–3 with respect to experimental 2–4. Greater learning in the 2.0 mA tDCS group compared with the 0.1 mA group during training, *F*(1,61) = 13.16, *p* = 0.0006; immediately after training, *F*(1,35) = 12.23, *p* = 0.001; and at the 1 h follow-up, *F*(1,35) = 11.09, *p* = 0.002.
**Coffman et al.,** (2012) [37]	Sample size: 55; Population: healthy participants; Age: *M* = 23.7; Sex: 22 females, 33 males.	Cognitive mechanisms underlying anodal tDCS-enhanced performance in a VR target-detection task.	*Two-arm, mixed* •Experiment 1 (*n* = 36) Experimental 1: anodal tDCS + VR (*n* = 13) Experimental 2: sham tDCS + VR (*n* = 23) •Experiment 2 (*n* = 19) Experimental 3: anodal tDCS + VR (*n* = 9) Experimental 4: sham tDCS + VR (*n* = 10)	A 1 h VR training session combined with active or sham tDCS.	JVC-DLA Multimedia projector (ModelDLA-SX200-NLG)JVC Kenwood Corporation, Yokohama, Japan)	•Region: IFC •Position: Anode F10, cathode was placed on the subject’s left upper arm. •Application: 30 min experimental 2 and 4 at 0.1 mA; experimental 1 and 3 at 2.0 mA. •Electrode dimensions/area (cm^2^): Saline sponge electrode/11.	•Testing stimuli: images containing objects •Hidden object detection accuracy •signal detection sensitivity (d′)	**Between-group results:** **↑** Improvement signal detection in experimental 1 and 3 with respect to experimental 2 and 4 (F = 21.003 *p* = 0.0003).
**Corrêa et al.,** (2023) [30]	Sample size: 57; Population: healthy older women; Age: 60 to 80; Sex: 57 females.	Effects of tDCS combined with VGT on postural balance in healthy older women.	*RCT—Three groups* • Experimental 1: anodal tDCS + VGT (*n* = 19) • Experimental 2: sham tDCS + VGT (*n* = 19) • Control: VGT (*n* = 19)	Eight 20 min sessions over 4 weeks (2 sessions/week) of VGT concurrently with active or sham tDCS.	Sony VPL-DX120 (Sony Corporation, Tokyo, Japan)	•Region: dlPFC •Position: Anode F3, cathode at right supraorbital region. •Application: Experimental 1 and 2 at 2 mA for 20 min; control only for 60 s. •Electrode dimensions: Anodal (5 × 5 cm^2^), cathodal (5 × 7 cm^2^), sponge.	•MMSE •BDI •Mini-BESTest/postural balance	**Between-group results:** No significant effect.
**Ferrucci et al.,** (2019) [32]	Sample size: 40; Population: healthy participants; Age: *M* = 26.65; Sex: 24 females, 16 males.	Cerebellar tDCS influences spatial navigation using VR.	RCT—Two groups • Experimental 1: anodal tDCS + VR •Control: sham tDCS + VR	One 20 min tDCS session after VR encoding, followed by 30 min of VR retrieval task.	Oculus Rift DK2 (Oculus VR LLC, Irvine, CA, USA)	•Region: Cerebellum •Position: Anode on the median line 2 cm below the inion; cathode over the right deltoid muscle. •Application: Experimental 1, 2 mA/cm^2^ for 20 min; control, for 20 s. •Electrode dimensions/area (cm^2^): Rectangular sponge 6 × 7 cm^2^.	•RTs •CSSL	**Between-group results:** No significant effect.
**Freire-** **Santos et al.,** (2025) [28]	Sample size: 107; Population: university students; Age: 18 to 50; Sex: 81 females, 26 males.	Effects of VR-FM, tDCS, and their combination on attention and inhibitory control.	*RCT—Five groups:* •Experimental 1: anodal tDCS + VR-FM (*n* = 21) •Experimental 2: anodal tDCS + VR-MW *(n* = 22) •Experimental 3: sham tDCS + VR-FM (*n* = 21) •Experimental 4: sham tDCS + VR-MW (*n* = 22) •Control: no intervention (*n* = 21).	One 20 min session of concurrent VR stimulation and active or sham tDCS.	Oculus Rift S (Oculus VR LLC, Menlo Park, CA, USA).	•Region: Left dlPFC •Position: Anode at F3, cathode at F4. •Application: Experimental 1 and 2 at 2 mA for 20 min; experimental 3 and 4 at 0.3 mA for 20 min. •Electrode area (cm^2^): 25.	•EST •SART •DASS-21 •MAAS •TMT •DERS-SF •nsSCR	**Between-group results:** **↓** Reduced nsSCR (*p* = 0.014) in experimental 1 with respect to experimental 4 group (*F*(3,66) = 4.07, *p* = 0.010, η^2^ = 0.156).
**Hui et al.,** (2024) [18]	Sample size: 61; Population: university students; Age: 23 to 35; Sex: 32 females, 29 males.	HD-tDCS can enhance the efficacy of VRET.	*RCT—Two groups* •Experimental: anodal HD-tDCS + VRET (*n* = 30) •Control: sham HD-tDCS + VRET (*n* = 31)	Two sessions of an active or sham HD-tDCS lasting 20 min followed by 50 min of VRET.	Meta Quest 2 (Meta Platforms, Inc., Menlo Park, CA, USA)	•Region: mPFC •Position: Anode at FPZ, cathodes at AF7, AF8, F3, and F4. •Application: Experimental 1.5 mA for 20 min; sham 0 mA for 20 min. •Electrode dimensions: HD-tDCS 4 × 1 montage.	•AQ •HIQ •STAI-Y •BAI •SUDS	**Between-group results:** **↓** Reduced anxiety (AQ) (*F*(2.60) = 8.56; *p* = 0.001) and discomfort(d = 0.61; *p* = 0.024) in the experimental group compared to the control group.
**Shahbazi et al.,** (2024) [29]	Sample size: 36; Population: sedentary teenage girls; Age: 15 to 18; Sex: 36 females.	Effects of dual-site tDCS combined with VR games on motor coordination in sedentary adolescent girls.	*RCT—Three groups* • Experimental 1: anodal tDCS + VR (*n* = 12) • Experimental 2: sham-tDCS + VR (*n* = 12) • Control: no-treatment (*n* = 12)	Twelve sessions over 4 weeks (3 sessions/week). Each session consisted of 20 min of active or sham tDCS followed by 60 min of VR.	Xbox 360 and Kinect Xbox 360 (Microsoft Corporation, Redmond, WA, USA)	•Region: M1 and dlPFC •Position: Two anodes at C3 and F3, two cathodes at AF4 and one centered between Fpz and AFz. •Application: Experimental 1 at 2 mA for 20 min; experimental 2 at 2 mA only for 30 s. •Electrode dimensions/area (cm^2^): Electrodes, two sponge anodes (5 × 4 cm^2^; 20 cm^2^), and two cathodes (9 × 4; 36 cm^2^).	•IPAQ •Automatic Mirror Trace (EHC) •Two-Arm Coordination Test device (BC)	**Between-group results:** ↑ Improved EHC in experimental 1 and 2 vs. control at post-intervention and 2-week follow-up (all *p*s < 0.001); experimental 1 > experimental 2 at follow-up (*p* = 0.024, *d* = 1.04). ↑ Improved BC in experimental 1 and 2 vs. control at post-intervention and 2-week follow-up (all *p*s < 0.001); experimental 1 > experimental 2 at follow-up (*p* < 0.001, *d* = 2.30).
**Yang et al.,** (2023) [38]	Sample size: 10; Population: healthy participants; Age: *M* = 24.3, *DS* = 1.5; Sex: 10 males.	Effects of anodal HD-tDCS on sustained attention in VR.	*Repeated measurements design:* • Experimental phase: anodal HD-tDCS + VR • Control phase: sham HD-tDCS + VR	One session (20 min) of active or sham tDCS followed by a 30 min 3D Go/No-go VR task. The two conditions (active or sham tDCS) were separated by a 2 h rest period.	Oculus Quest Oculus VR LLC, Menlo Park, CA, USA)	•Region: rVLPFC •Position: Anode at FC6, cathode in 4 × 1 ring at F4, F8, C4, T8. •Application: Experimental 1 mA for 10.5 min (15 s fade-in/out); control 1 mA during both the first and last 30 s. •Electrode dimensions/area (cm^2^): HD-tDCS 4 × 1 montage.	•Go-No-go task •EEG (ERPs) •Self-reports of attentional level	**Within-group results:** **↑** Improved perceived attention during experimental intervention compared to control intervention (z = −2.81; *p* = 0.005). **↑** Improved accuracy in experimental intervention compared to control intervention (z = −2.80; *p* = 0.005). **↑** Improved reaction time in experimental intervention compared to control intervention (z = 1.99, *p* = 0.047).
**Takeuchi et al.,** (2018) [39]	Sample size: 20; Population: healthy participants; Age: *M* = 21.5, *SD* = 1.1; Sex: 11 females, 9 males.	Effects of tDCS cortical modulation on VR-related sickness.	*Repeated measurements design.* •Experimental phase 1: anodal tDCS + VR •Experimental phase 2: cathodal tDCS + VR •Control phase: sham tDCS+ VR	One 15 min tDCS session, followed by 15 min of VR roller-coaster immersion.	HTC VIVE HTC Corporation, Taoyuan City, Taiwan)	•Region: TPJ •Position: Anode CP6, cathode Cz. •Application: Anodal and cathodal condition at 1.5 mA for 15 min; sham condition 1.5 mA for 30 s. • Electrode dimensions/area (cm^2^): Gel-sponge/25 cm^2^.	• SSQ •Heart rate • COP baseline	**Within-group results:** **↓** Reduced the disorientation score at post-VR (*F*(4,76) = 4.90) in experimental 1 intervention with respect to control intervention (*p* = 0.042) and experimental 2 intervention (*p* = 0.040). **↓** Reduced the change in COP length at post-VR (*F*(4,76) = 4.229) in experimental 1 intervention than in both control intervention (*p* = 0.049) and experimental 2 intervention (*p* = 0.007).

Legend: AQ: Acrophobia Questionnaire; ATHQ: Attitude Towards Heights Questionnaire; BAI: Beck Anxiety Inventory; BC: Bimanual Coordination; BDI: Beck Depression Inventory; CGI: Clinical Global Impression; COP: Center of Pressure; CSSL: Corsi Supra-Span Learning; CS: Cybersickness; DASS-21: Depression Anxiety and Stress Scale; DERS-SF: Difficulties in Emotion Regulation Scale-Short Form; EDA: Electro-Dermal Activity; EEG: Electroencephalography; EHC: Eye–Hand Coordination; EMG: Electro-myogram; ERPs: Event-Related Potentials; EST: Emotional Stroop; FM: Focused Mindfulness Meditation; HD-tDCS: High-Definition Transcranial Direct Current Stimulation; HIQ: Heights Interpretation Questionnaire; IPAQ: International Physical Activity Questionnaire; IPQ: Igroup Presence Questionnaire; MAAS: Mindful Attention and Awareness Scale; MMSE: Mini-Mental State Examination; M1: Primary Motor Cortex; MEC-SPQ: Spatial Presence Questionnaire; mPFC: Medial Prefrontal Cortex; MW: Mind-Wandering; NF: Neurofeedback; nsSCR: Non-Specific Skin Conductance Response; RCT: Randomized Control Trial; RTs: Simple Visual Reaction Times; rVLPFC: Ventrolateral Prefrontal Cortex; SAM: Self-Assessment Manikin; SART: Sustained Attention to Response Task; SCL: Skin Conductance Level; SCR: Skin Conductance Responses; SSQ: Simulator Sickness Questionnaire; STAI-Y: State–Trait Anxiety Inventory Form Y; SUDs: Subjective Units of Distress; SUDs: Subjective Units of Discomfort; TMT: Trail Making Test A and B; VGT: Video Game Training; vHIQ: Visual Height Intolerance Questionnaire; vmPFC: Ventromedial Prefrontal Cortex; VRET: Virtual Reality Exposure Therapy.

## Data Availability

The data extracted and analyzed during this systematic review are available from the corresponding author upon reasonable request.
